# Operative Treatment of Irreducible Dislocation of the Shoulder, Recent or Old, Simple or Complicated

**Published:** 1897-06

**Authors:** Edmond Souchon

**Affiliations:** Professor of Anatomy and Clinical Surgery, Tulane University, New Orleans, La.


### OPERATIVE TREATMENT OF IRREDUCIBLE DISLOCATIONS OF THE SHOULDER, RECENT OR OLD, SIMPLE OR COMPLICATED.

BY EDMOND SOUCHON, M. D., PROFESSOR OF ANATOMY AND CLINICAL SURGERY, TULANE UNIVERSITY, NEW ORLEANS, LA.

The writer considers all the forms and varieties of irreducible dislocations of the shoulder, and studies for each one the operations performed, the difficulties and complications during the operation, the complications after the operation, the results immediate, the results remote, and formulates the conclusions as to the advantages and disadvantages of each. The study is based on 140 operated cases. The profound silence of text-books, and also of special books on dislocations, in regard to this most important subject, renders this study most imperative and timely.

All *irreducible dislocations recent, simple, or complicated, with fracture,* that were operated by reduction or resection have given good results with two exceptions, in which death does not seem to be due truly to the operation alone, but to complications of shock, unusually severe at that.

*Irreducible dislocations, old, simple and forward,* operated by *resection* through an *anterior incision* are the most frequent—56 cases against 33 by arthrotomy and reduction. Results remote show a great mortality in resection from injuries to the vessels mostly and from sepsis, but this is avoidable with special care. The fatalities in reductions are due to sepsis now preventable. The disadvantages of reduction are necrosis of the cartilage and of the bone of the head, calling later for sequestrotomy and resection. This probably is due to the greater dissections and denudations of the head and surgical neck necessary to reduce than to simply resect the head. Reduction is the more desirable operation of the two because it preserves the head and all the movements depending therefrom, but the necrotic consequences are serious drawbacks, as is also anchylosis following sometimes the reduction. It should not be resorted to unless it can be done easily, without too extensive dissections, although it may be necessary to use hooks, levers, and some curetting of the cup, as the cases reported show. The duration of the dislocation is immaterial; it is the condition of the parts that is all important. Some recent irreducible dislocations have given as much if not more trouble to reduce than dislocations of months' standing.

The anterior incision is the route in all forward dislocations; almost all *forward cases operated by the axillary route* and *the posterior incision* have been unfavorable.

Cases reported as *operated by subcutaneous sections of fibrous bands, of tendons, of muscles,* and by *osteotomy,* have given good

*Paper Read before the American Surgical Association, Washington, D. C., May 1897.

results, but they are so few. It seems extraordinary that surgeons have not employed these methods oftener; perhaps they have, but having failed, have not reported the cases. In considering the extent and the density of the tissues binding down the head and surgical neck to the surrounding parts, as reported by operators, it is a wonder that these methods should succeed except in very selected loose cases.

*Irreducible dislocations, old and downward*, four in number, have all been treated by the axillary incision with the resection—*i. e.*, removal of the fractured head, more or less loose in the axilla, with favorable termination. In one case, however, the head was "pegged back" and reduced with a good result. One downward dislocation (Despre's) was treated by osteoclasia, but it was a failure, no false joint forming. Yet there are cases on record of forward dislocation in which the bone was fractured near the head or through the surgical neck during efforts at reduction, and which yielded a fair enough result.

*Irreducible dislocations, old and backward*, in the adult, have been reported twice. They were operated by resection with a very ordinary result.

*Irreducible dislocations, old, upward and operated*, have not been found on record.

*Irreducible dislocations, congenital*, have been operated several times; they were old, backward dislocations. Two cases were operated by reduction; one case died, the other had to have sequestra removed, and then did well. Three cases were operated by resection, two with good results; the third one is not stated.

*Irreducible old dislocations in young subjects* and in *old subjects* are duly considered, also old *dislocations double*, *i. e.*, of both shoulders; also spontaneous or *pathological*, paralytic irreducible old dislocations.

The *forms and varieties* due to *complications* accompanying irreducible old dislocatious are fully treated, and also the forms and varieties due to relapses or *recurrences* and to the *sequels of the operations* performed for irreducible dislocations, old, simple, or complicated.—*New Orleans Medical and Surgical Journal.*

